# Suicidality in Treatment-Resistant Depression Patients

**DOI:** 10.1192/j.eurpsy.2023.704

**Published:** 2023-07-19

**Authors:** E. B. Lyubov, N. D. Semenova

**Affiliations:** ^1^Suicidology; ^2^Psychological Counselling, Moscow Research Institute of Psychiatry, Moscow, Russian Federation

## Abstract

**Introduction:**

Depression and treatment-resistant depression (RD) are associated with suicidal behavior (SB) at a higher rate.

**Objectives:**

1) determination prevalence of RD in district outpatient psychiatric сlinics (i.e., dispensaries) and the socio-demographic characteristics of RD patients with SB.

**Methods:**

In this multicenter (3 sites), retrospective, observational epidemiological study, patients (n=148) with diagnoses F 30-39 (ICD-10) were recruited in 2020. Patients (n=22) were assessed for RD, defined as failure to respond to ≥ two antidepressant medications of adequate dose and duration for at least three months.

**Results:**

The prevalence of depression is ≤ 2% of the outpatient population. RD prevalence ˜15%. SB (i.e., suicidal attempts) was noted in every fifth (n=5) for the index year. SB patients differed in the following typical features: a woman (82%) mean age, 46.8 years with long-term (≥ 10 years) depression and annual hospitalizations for three years (61.4 days per patient), and with suicidal attempts (repeated within the last two years) through (80%) self-poisoning with psychotropic drugs. Nobоdy worked, and everyone was divorced.

**Conclusions:**

On the background of ubiquitous underdiagnosis of depressive disorders in routine practice, RD patients with SP represent a high-resource users group with combined clinical and social problems requiring pharmacotherapy and target psychosocial rehabilitation.

**Disclosure of Interest:**

None Declared

